# Phosphate solubilizing microbes: sustainable approach for managing phosphorus deficiency in agricultural soils

**DOI:** 10.1186/2193-1801-2-587

**Published:** 2013-10-31

**Authors:** Seema B Sharma, Riyaz Z Sayyed, Mrugesh H Trivedi, Thivakaran A Gobi

**Affiliations:** Department of Earth and Environmental Science, KSKV Kachchh University, Mundra Road, Bhuj, Gujarat 370 001 India; Department of Microbiology, PSGVP Mandal’s Arts, Science and Commerce College, Shahada, Maharashtra 425409 India; Gujarat Institute of Desert Ecology, Bhuj, Gujarat India

**Keywords:** Soil phosphorus, PSM, P solubilization, Biodiversity, Biofertilizers

## Abstract

Phosphorus is the second important key element after nitrogen as a mineral nutrient in terms of quantitative plant requirement. Although abundant in soils, in both organic and inorganic forms, its availability is restricted as it occurs mostly in insoluble forms. The P content in average soil is about 0.05% (w/w) but only 0.1% of the total P is available to plant because of poor solubility and its fixation in soil (Illmer and Schinner, Soil Biol Biochem 27:257-263, 1995). An adequate supply of phosphorus during early phases of plant development is important for laying down the primordia of plant reproductive parts. It plays significant role in increasing root ramification and strength thereby imparting vitality and disease resistance capacity to plant. It also helps in seed formation and in early maturation of crops like cereals and legumes. Poor availability or deficiency of phosphorus (P) markedly reduces plant size and growth. Phosphorus accounts about 0.2 - 0.8% of the plant dry weight.

To satisfy crop nutritional requirements, P is usually added to soil as chemical P fertilizer, however synthesis of chemical P fertilizer is highly energy intensive processes, and has long term impacts on the environment in terms of eutrophication, soil fertilility depletion, carbon footprint. Moreover, plants can use only a small amount of this P since 75–90% of added P is precipitated by metal–cation complexes, and rapidly becomes fixed in soils. Such environmental concerns have led to the search for sustainable way of P nutrition of crops. In this regards phosphate-solubilizing microorganisms (PSM) have been seen as best eco-friendly means for P nutrition of crop. Although, several bacterial (*pseudomonads and bacilli*) and fungal strains (*Aspergilli* and *Penicillium*) have been identified as PSM their performance under *in situ* conditions is not reliable and therefore needs to be improved by using either genetically modified strains or co-inoculation techniques. This review focuses on the diversity of PSM, mechanism of P solubilization, role of various phosphatases, impact of various factors on P solubilization, the present and future scenario of their use and potential for application of this knowledge in managing a sustainable environmental system.

**Electronic supplementary material:**

The online version of this article (doi:10.1186/2193-1801-2-587) contains supplementary material, which is available to authorized users.

## Introduction

Phosphorus is the most important key element in the nutrition of plants, next to nitrogen (N). It plays an important role in virtually all major metabolic processes in plant including photosynthesis, energy transfer, signal transduction, macromolecular biosynthesis and respiration (Khan et al. 2010) and nitrogen fixation in legumes (Saber et al. 2005). Although P is abundant in soils in both inorganic and organic forms, it is a major limiting factor for plant growth as it is in an unavailable form for root uptake. Inorganic P occurs in soil, mostly in insoluble mineral complexes, some of them appearing after frequent application of chemical fertilizers. These insoluble, precipitated forms cannot be absorbed by plants (Rengel and Marschner 2005). Organic matter is also an important reservoir of immobilized P that accounts for 20–80% of P in soils (Richardson 1994). Only 0.1% of the total P exists in a soluble form available for plant uptake (Zhou et al. 1992) because of its fixation into an unavailable form due to P fixation. The term P fixation is used to describe reactions that remove available phosphate from the soil solution into the soil solid phase (Barber 1995). There are two types of reactions (a) phosphate sorption on the surface of soil minerals and (b) phosphate precipitation by free Al^3+^ and Fe^3+^ in the soil solution (Havlin et al. 1999). The soils that exhibit highest P fixation capacity occupy 1,018 million hectares (ha) in the tropics (Sanchez and Logan 1992). It is for this reason that soil P becomes fixed and available P levels have to be supplemented on most agricultural soils by adding chemical P fertilizers, which not only represent a major cost of agricultural production but also impose adverse environmental impacts on overall soil health and degradation of terrestrial, freshwater and marine resources (Tilman et al. 2001). Thus, increased P levels have been identified as a main factor for eutrophication of surface waters that may lead to algal blooms (Schindler et al. 2008). The repeated and injudicious applications of chemical P fertilizers, leads to the loss of soil fertility (Gyaneshwar et al. 2002) by disturbing microbial diversity, and consequently reducing yield of crops. The long-term effect of different sources of phosphate fertilizers on microbial activities includes inhibition of substrate-induced respiration by streptomycin sulphate (fungal activity) and actidione (bacterial activity) and microbial biomass carbon (C) (Bolan et al. 1996). Similarly, the application of triple superphosphate (94 kg/ ha) has shown a substantial reduction in microbial respiration and metabolic quotient (qCO_2_) (Chandini and Dennis 2002).

Moreover the efficiency of applied P fertilizers in chemical form rarely exceeds 30% due to its fixation, either in the form of iron/aluminium phosphate in acidic soils (Norrish and Rosser 1983) or in the form of calcium phosphate in neutral to alkaline soils (Lindsay et al. 1989). It has been suggested that the accumulated P in agricultural soils would be sufficient to sustain maximum crop yields worldwide for about 100 years if it were available (Khan et al. 2009a, b, c). A major characteristic of P biogeochemistry is that only 1% of the total soil P (400–4,000 kg P/ ha in the top 30 cm) is incorporated into living plant biomass during each growing season (10–30 kg P/ha), reflecting its low availability for plant uptake (Blake et al. 2000; Quiquampoix and Mousain 2005). Furthermore P is a finite resource and based on its current rate of use, it has been estimated that the worlds known reserves of high quality rock P may be depleted within the current century (Cordell et al. 2009). Beyond this time the production of P based fertilizers will require the processing of lower grade rock at significantly higher cost (Isherwood 2000). The realization of all these potential problems associated with chemical P fertilizers together with the enormous cost involved in their manufacture, has led to the search for environmental compatible and economically feasible alternative strategies for improving crop production in low or P-deficient soils (Zaidi et al. 2009). The use of microbial inoculants (biofertilisers) possesing P-solubilizing activities in agricultural soils is considered as an environmental-friendly alternative to further applications of chemical based P fertilizers.

### Constraints in using phosphate fertilizers

There is global concern about the energy and costs involved in mining the rock phosphate and its transport from manufacturing sites to farm crop fields. Mining phosphate minerals and spreading P fertilizers over the landscape is neither eco-friendly, economically feasible nor it is sustainable and it poses following constraints (i) emission of the fluorine as the highly volatile and poisonous HF gas, (ii) disposal of gypsum and (iii) accumulation of Cd and other heavy metals in soil and possibly crops as a result of repetitive use of P fertilizers.

At present mining rate (about 7,100 million tones/annum), reserve will be depleted in about 500–600 years. In India, deposits of sufficiently enriched phosphatic rocks are limited and hence it imports 2 million tons of rock phosphate annually. About 98% of cropland in India is deficient in available forms of soil phosphorus and only 1-9% has high phosphorus status. Intensive cropping pattern during this green and white revolution has also resulted in widespread deficiency of phosphorus. Although various amendments are available for management of P in different soil, all are costlier and practically difficult. Thus, even if the total soil P is high and also if P fertilizers are applied regularly, pH dependent chemical fixation determines the quantity of available P. The holistic P management involves a series of strategies involving manipulation of soil and rhizosphere processes, development of P efficient crops and improving P recycling efficiency. Microbial mediated P management is an ecofriedly and cost effective approach for sustainable development of agricultural crop.

Microorganisms are an integral component of the soil P cycle and are important for the transfer of P between different pools of soil P. Phosphate Solubilzing Microorganisms (PSM) through various mechanisms of solubilization and mineralisation are able to convert inorganic and organic soil P respectively (Khan et al. 2009a) into the bioavailable form facilitating uptake by plant roots. It is important to determine the actual mechanism of P solubilisation by PSM for optimal utilization of these microorganisms in varied field conditions. Hence it is imperative to better understand the plant-soil-microbial P cycle with the aim of reducing reliance on chemical P fertilizers. This has led to increased interest in the harnessing of microorganisms to support P cycling in agroecosystems.

### Occurrence and isolation of PSM

Solubilization of insoluble P by microorganisms was reported by Pikovskaya (1948). During the last two decades knowledge on phosphate solubilizing microorganisms increased significantly (Richardson 2001; Rodriguez and Fraga 1999). Several strains of bacterial and fungal species have been described and investigated in detail for their phosphate-solubilizing capabilities (Glick 1995; He et al. 1997). Typically such microorganisms have been isolated using cultural procedures with species of *Pseudomonas* and *Bacillus* bacteria (Illmer and Schinner 1992) and *Aspergillus* and *Penicillium* fungi being predominant (Wakelin et al. 2004). These organisms are ubiquitous but vary in density and mineral phosphate solubilizing (mps) ability from soil to soil or from one production system to another. In soil P solubilizing bacteria constitute 1-50% and fungi 0.1-0.5% of the total respective population. They are generally isolated from rhizosphere and nonrhizosphere soils, rhizoplane, phyllosphere, and rock P deposit area soil and even from stressed soils using serial plate dilution method or by enrichment culture technique (Zaidi et al. 2009).

The concentration of iron ore, temperature, and C and N sources greatly influence the P-solubilizing potentials of these microbes. Among the various nutrients used by these microorganisms, ammonium salts has been found to be the best N source followed by asparagine, sodium nitrate, potassium nitrate, urea and calcium nitrate (Ahuja et al. 2007). Since 1948, when Pikovskaya suggested that microbes could dissolve non-readily available forms of soil P and play an important role in providing P to plants, numerous methods and media, such as Pikovskaya (Pikovskaya 1948), bromophenol blue dye method (Gupta et al. 1994) and National Botanical Research Institute P (NBRIP) medium (Nautiyal 1999) have been proposed. The source of insoluble phosphate in the culture media to isolate PSM is a major issue of controversy regarding the isolation of PSM in true sense. Commonly used selection factor for this trait, tricalcium phosphate (TCP), is relatively weak and unreliable as a universal selection factor for isolating and testing phosphate-solubilizing microorganisms (PSM) for enhancing plant growth. The use of TCP usually yields many (up to several thousand per study) isolates of “supposed” PSM. When these isolates are further tested for direct contribution of phosphorus to the plants, only a very few are true PSM. Other compounds are also tested, but on a very small scale. These phosphates, mainly iron/aluminium phosphate and several calcium phosphates are even less soluble than TCP in water. Because soils greatly vary in pH and several chemical properties, it appears that there is no metal-Phosphate compound that can serve as the universal selection factor for PSM. Here multiple sources of insoluble phosphate are recommended. The selection of the metal-Phosphate candidates for potential PSM will depend on the type of soil (alkaline, acidic, or organic-rich) where the PSM will be used. Adding calcium phosphate compounds (including rock phosphates) for alkaline soils, iron/aluminium phosphate compounds for acidic soils, and phytates for soils rich in organic P is suggested Bashan et al. 2013a, b.

Both bacterial and fungal strains exhibiting P solubilizing activity are detected by the formation of clear halo (a sign of solubilization) around their colonies. Production of a halo on a solid agar medium should not be considered the sole test for P solubilization. When colonies grow without a halo after several replacements of the medium, an additional test in liquid media to assay P dissolution should be performed and the few isolates that are obtained after such rigorous selection should be further tested for abundant production of organic acids and the isolates complying with these criteria should be tested on a model plant as the ultimate test for potential P solubilization (Bashan et al. 2013a). The viable microbial preparations possessing P-solubilizing activity are generally termed as microphos (Zaidi et al. 2009). The phosphate-solubilizing microbes showing greater solubilization (both qualitatively and quantitatively) of insoluble P under in vitro conditions are selected for field trials prior to production in bulk for ultimate transmission as a biofertiliser.

Once a potential isolate is identified, it must be further tested for direct contribution to P plant nutrition and not necessarily to general growth promotion, as commonly done because promotion of growth, even by PSB, can be the outcome of other mechanisms. (Bashan et al. 2013a) and abilty to solubilise P is not necessarily correlated with the ability to promote plant growth (Collavino et al. 2010).

**Figure 1:**
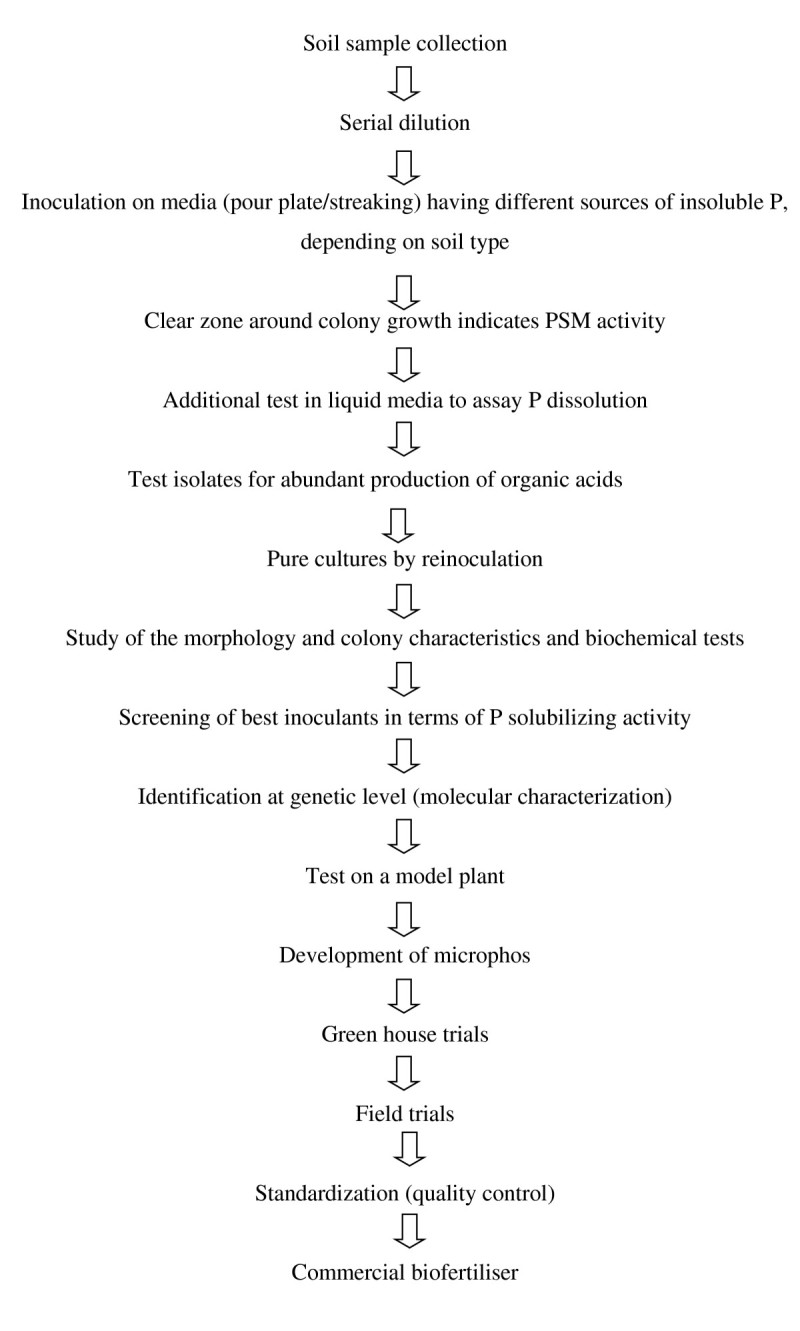
**Protocol for isolation and development of effective inoculants of PSM based biofertiliser.**

The production of biofertilizer and its acceptance by farming communities are closely linked. For uptake by farmers, quality management is essential and must be performed consistently in order to supply reliable and contaminant-free bio products. As far as *in vitro* field trials are concerned the establishment and performance of these PSM inoculate developed in laboratory is largely hampered by environmental variables including salinity, pH, moisture, temperature and climatic conditions of the soil. Moreover it is also known that inocula developed from a particular soil fail to function as effectively in soils having different properties (Rodriguez and Fraga 1999). Hence there is a need to study PSM activity in correlation with these factors before PSM application as a biofertiliser. Protocol for isolation and effective inoculants development of PSM based biofertiliser has been shown in Figure 1.

### Biodiversity of P solubilizers

A substantial number of microbial species exhibit P solubilization capacity; these include bacteria, fungi, actinomycetes and even algae. In addition to *Pseudomonas* and *Bacillus*, other bacteria reported as P-solubilizers include *Rhodococcus*, *Arthrobacter, Serratia, Chryseobacterium, Gordonia, Phyllobacterium, Delftia sp.* (Wani et al. 2005; Chen et al. 2006), *Azotobacter* (Kumar et al. 2001), *Xanthomonas* (De Freitas et al. 1997), *Enterobacter, Pantoea, and Klebsiella* (Chung et al. 2005), *Vibrio proteolyticus, Xanthobacter agilis* (Vazquez et al. 2000). Furthermore, symbiotic nitrogenous *rhizobia*, which fix atmospheric nitrogen into ammonia and export the fixed nitrogen to the host plants, have also shown PS activity (Zaidi et al. 2009) For instance, *Rhizobium leguminosarum bv. Trifolii* (Abril et al. 2007), and *Rhizobium* species nodulating *Crotalaria* species (Sridevi et al. 2007) improved plant P-nutrition by mobilizing inorganic and organic P. Various PS bacteria have also been isolated from stressed environments for example the halophilic bacteria *Kushneria sinocarni* isolated from the sediment of Daqiao saltern on the eastern coast of China, which may be useful in salt affected agricultural soils (Zhu et al. 2011).

In soil, P-solubilizing fungi constitute about 0.1–0.5% of total fungal populations (Kucey 1983). Moreover, P-solubilizing fungi do not lose the P dissolving activity upon repeated sub culturing under laboratory conditions as occurs with the P-solubilizing bacteria (Sperber 1958a, b; Kucey 1983). Moreover, fungi in soils are able to traverse long distances more easily than bacteria and hence, may be more important to P solubilization in soils (Kucey 1983). Generally, the P-solubilizing fungi produce more acids than bacteria and consequently exhibit greater P-solubilizing activity (Venkateswarlu et al. 1984). Among filamentous fungi that solubilize phosphate, the genera *Aspergillus* and *Penicillium* (Fenice et al. 2000; Khan and Khan 2002; Reyes et al. 1999, 2002) are the most representative although strains of *Trichoderma* (Altomare et al. 1999) and *Rhizoctonia solani* (Jacobs et al. 2002) have also been reported as P solubilizers. A nematofungus *Arthrobotrys oligospora also* has the ability to solubilize phosphate *in vivo* as well as *in vitro* (Duponnois et al. 2006). Among the yeasts, only a few studies have been conducted to assess their ability to solubilize phosphate these include *Yarrowia lipolytica* (Vassilev et al. 2001), *Schizosaccharomyces pombe* and *Pichiafermentans*. As more studies are conducted, a wider diversity of phosphate-solubilizing filamentous fungi are expected to be described. Of those identified, many are commonly found in agricultural soils such as *Penicillium sp.*, *Mucor sp.* and *Aspergillus sp.* which has been shown to increase plant growth by 5–20% after inoculation (Gunes et al. 2009).

The P-solubilizing ability of actinomycetes has attracted interest in recent years because this group of soil organisms is not only capable of surviving in extreme environments (e.g. drought, fire.) but also possess other potential benefits (e.g. production of antibiotics and phytohormone-like compounds) that could simultaneously benefit plant growth (Fabre et al. 1988; Hamdali et al. 2008a, b). A study by Hamdali et al. (2008a) has indicated that approximately 20% of actinomycetes can solubilize P, including those in the common genera *Streptomyces* and *Micromonospora*. A partial list of PSM including various groups is given in Table 1.

**Table 1:** **Biodiversity of PSM**

**Bacteria**	*Alcaligenes sp., Aerobactor aerogenes, Achromobacter sp., Actinomadura oligospora, Agrobacterium sp., Azospirillum brasilense, Bacillus sp., Bacillus circulans, B.cereus, B.fusiformis, B. pumils, B. megaterium, B. mycoides, B. polymyxa, B. coagulans B,.chitinolyticus, B. subtilis, Bradyrhizobium sp., Brevibacterium sp., Citrobacter sp., Pseudomonas sp., P putida, P. striata, P. fluorescens, P. calcis, Flavobacterium sp., Nitrosomonas sp., Erwinia sp., Micrococcus sp., Escherichia intermedia, Enterobacter asburiae, Serratia phosphoticum, Nitrobacter sp., Thiobacillus ferroxidans, T. thioxidans, Rhizobium meliloti, Xanthomonas sp.*
**Fungi**	*Aspergillus awamori, A. niger, A. tereus, A. flavus, A. nidulans, A. foetidus, A. wentii. Fusarium oxysporum, Alternaria teneius, Achrothcium sp. Penicillium digitatum, P lilacinium, P balaji, P. funicolosum, Cephalosporium sp. Cladosprium sp., Curvularia lunata, Cunnighamella, Candida sp., Chaetomium globosum, Humicola inslens, Humicola lanuginosa, Helminthosporium sp., Paecilomyces fusisporous, Pythium sp., Phoma sp., Populospora mytilina, Myrothecium roridum, Morteirella sp., Micromonospora sp., Oideodendron sp., Rhizoctonia solani, Rhizopus sp., Mucor sp., Trichoderma viridae, Torula thermophila, Schwanniomyces occidentalis, Sclerotium rolfsii.*
**Actinomycetes**	*Actinomyces,, Streptomyces.*
**Cyanobacteria**	*Anabena sp., Calothrix braunii, Nostoc sp., Scytonema sp.,*
**VAM**	*Glomus fasciculatum.*

In addition to bacteria, fungi and actinomycetes, algae such as cyanobacteria and mycorrhiza have also been reported to show P solubilization activity.The interactive effects of arbuscular mycorrhizal fungi (AMF) and rhizobacteria on the growth and nutrients uptake of Sorghum bicolor were studied in acid and low availability phosphate soil. The microbial inocula consisted of the AMFs *Glomus manihotis* and *Entrophospora colombiana*, PSB *Pseudomonas* sp., results indicated that the interaction of AMF and the selected rhizobacteria has a potential to be developed as biofertilizers in acid soil. The potential of dual inoculation with AMF and rhizobacteria needs to be further evaluated under different crop and agroclimatic conditions, particularly in the field (Widada et al. 2007).

Hence the studies have shown that the diversity of the PSM’s is highly varied in different ecological niches and there is ample scope to identify many new potent isolates from varied environments in coming times.

### Mechanism of P-solubilization by PSM

In a review of P chemistry in soils, Sims and Pierzynski (2005) identified the major processes of the soil P cycle that affect soil solution P concentrations as(1) dissolution–precipitation (mineral equilibria), (2) sorption–desorption (interactions between P in solution and soil solid surfaces), and (3) mineralization–immobilization (biologically mediated conversions of P between inorganic and organic forms).

The main P solubilization mechanisms employed by soil microorganisms include: (1) release of complexing or mineral dissolving compounds e.g. organic acid anions, siderophores, protons, hydroxyl ions, CO_2_, (2) liberation of extracellular enzymes (biochemical P mineralization) and (3) the release of P during substrate degradation (biological P mineralization) (McGill and Cole 1981). Therefore, microorganisms play an important role in all three major components of the soil P cycle (i.e. dissolution–precipitation, sorption–desorption, and mineralization–immobilization). Additionally these microorganisms in the presence of labile C serve as a sink for P, by rapidly immobilizing it even in low P soils; therefore PSM become a source of P to plants upon its release from their cells. Release of P immobilized by PSM primarily occurs when cells die due to changes in environmental conditions, starvation or predation. Environmental changes, such as drying–rewetting or freezing–thawing, can result in so-called flush-events, a sudden increase in available P in the solution due to an unusually high proportion of microbial cell lysis (Butterly et al. 2009). (Grierson et al. 1998) found that about 30–45% of microbial P (0.8–1 mg kg^-1^) was released in a sandy spodosol in an initial flush after drying–rewetting cycles within the first 24 hour.

**A) InorganicPsolubilization:** by P-solubilizing microorganisms occurs mainly by organic acid production (Table 2), either by: (i) lowering the pH, or (ii) by enhancing chelation of the cations bound to P (iii) by competing with P for adsorption sites on the soil (iv) by forming soluble complexes with metal ions associated with insoluble P (Ca, Al, Fe) and thus P is released. The lowering in pH of the medium suggests the **release of organic acids** by the P-solubilizing microorganisms (Whitelaw 2000; Maliha et al. 2004) via the direct oxidation pathway that occurs on the outer face of the cytoplasmic membrane (Zaidi et al. 2009). These acids are the product of the microbial metabolism, mostly by oxidative respiration or by fermentation of organic carbon sources (e.g., glucose) (Atlas and Bartha 1997; Trolove et al. 2003) or such organic acids can either directly dissolve the mineral P as a result of anion exchange of phosphate by acid anion or can chelate Fe, Al and Ca ions associated with P (Omar 1998).

**Table 2:** **Important PSM, their ecological niches and organic acids produced**

Organism	Ecological niche	Predominant acids produced	Reference
PSB	Soil and phosphate bearing rocks	ND(not determined)	Pikovskaya 1948
PSB	Bulk and rhizospheric soil	ND(not determined)	Gerretson 1948
*Escherichia freundii*	Soil	Lactic	Sperber 1958a, b
*Aspergillus niger, Penicillium sp.*	Soil	Citric, glycolic, succinic, gluconic, oxalic, lactic	Sperber 1958a, b
*Bacillus megaterium, Pseudomonas sp., Bacillus subtilus*	Rhizospheric soil	Lactic, malic	Taha et al. 1969
*Arthrobacter sp., Bascillus sp., Bacillus firmus B-7650*	Wheat and cowpea rhizosphere	Lactic, citric	Bajpai and Sundara Rao 1971
*Aspergillus sp., Penicillium sp., Chaetomiumnigricolor*	Lateritic soil	Oxalic, Succinic, Citric, 2-ketogluconic	Banik and Dey 1983
*A. japonicus, A. foetidus*	Indian Rock phosphate	Oxalic, citric, gluconic succinic, tartaric acid	Singal et al. 1994
*P. radicum*	rhizosphere of wheat roots,	Gluconic	Whitelaw et al. 1999
*Enterobacteragglomerans*	Wheat rhizosphere	Oxalic, citric	Kim et al. 1997
*Bacillus amyloliquefaciens, B. licheniformis, B. atrophaeus, Penibacillus macerans, Vibrio proteolyticus, xanthobacter agilis, Enterobacter aerogenes, E. taylorae, E. asburiae, Kluyvera cryocrescens, Pseudomonas aerogenes, Chryseomonas Luteola*	Mangrove ecosystem	Lactic, itaconic, isovaleric, isobutyric, acetic	Vazquez et al. 2000
*Penicillium rugulosum*	Venezuelan phosphate rocks	Citric, gluconic acid	Reyes et al. 2001
*Enterobacter intermedium*	Grass rhizosphere	2-ketogluconic	Hwangbo et al. 2003
*Aspergillus flavus, A . niger, Penicilliumcanescens*	stored wheat grains	Oxalic, citric, gluconic succinic	Maliha et al. 2004
*P.fluorescens*	Root fragments and rhizosphere of oil palm trees	Citric, malic, tartaric, gluconic	Fankem et al. 2006
*Aspergillus niger*	Tropical and subtropical soil	Gluconic, oxalic	Chuang et al. 2007
*P.trivialis*	rhizosphere of *Hippophaer hamnoides* growing in the cold deserts of Lahaul and Spiti in the trans-Himalayas	Lactic, formic	Vyas and Gulati 2009
*B.pumilus var.2;B.subtilisvar.2;Actinomadura oligospora; Citrobacter sp.*	Giant Cardon cactus (P.pringlei) growing in ancient lava	Gluconic, Propionic, Isovaleric, Heptonic, Caproic, Isocaproic, Formic, Valeric, Succinic, Oxalic, Oxalacetic, Malonic.	Puente et al. 2004a
*B.pumilus CHOO8A; B.fusiformis*	Cholla cactus(Opuntia Cholla)		Puente et al. 2004a
*Bacillus sp. SENDO 6 and*	Giant Cardon cactus (P.pringlei)	Gluconic, Propionic, Isovaleric, Formic, Succinic, Lactic.	Puente et al. 2009a, b
*Pseudomonas putida M5TSA, Enterobacter sakazakii M2PFe, and Bacillus megaterium M1PCa*	Wild cactus Mammillaria fraileana		Lopez et al. 2011

The monovalent anion phosphate H_2_PO_4_^-^ is a major soluble form of inorganic phosphate, which usually occurs at lower pH. However as the pH of the soil environment increases the divalent and trivalent forms of Pi (HPO_4_^-2^ and HPO_4_^-3^ respectively) occur. Thus, the synthesis and discharge of organic acid by the PSM strains into the surrounding environment acidify the cells and their surrounding environment that ultimately lead to the release of P ions from the P mineral by H^+^ substitution for the cation bound to phosphate (Goldstein 1994). The prominent acids released by PSM in the solubilization of insoluble P are gluconic acid (Di-Simine et al. 1998; Bar-Yosef et al. 1999), oxalic acid, citricacid (Kim et al. 1997), lactic acid, tartaric acid, aspartic acid (Venkateswarlu et al. 1984). Evidence from an abiotic study using HCl and gluconic acid to solubilize P also indicated that chelation of Al^3+^ by gluconic acid may have been a factor in the solubilization of colloidal Al phosphate (Whitelaw et al. 1999). Organic acids produced by P-solubilizing microorganisms can be detected by high performance liquid chromatography and enzymatic methods (Parks et al. 1990; Whitelaw 2000). However, acidification does not seem to be the only mechanism of solubilization, as the ability to reduce the pH in some cases did not correlate with the ability to solubilize mineral P (SubbaRao 1982). Altomare et al. (1999) investigated the capability of the plant-growth promoting and biocontrol fungus *T. harzianum T-22* to solubilize in vitro insoluble minerals including rock phosphate. Organic acids were not detected in the culture filtrates and hence, the authors concluded that acidification was probably not the major mechanism of solubilization as the pH never fell below 5. The phosphate solubilizing activity was attributed both to chelation and to reduction processes. Although, organic acid has been suggested as the principal mechanism of P solubilization, the solubilization of insoluble P **by inorganic acid** (e.g. HCl) has also been reported, although HCl was able to solubilize less P from hydroxyapatite than citric acid or oxalic acid at same pH (Kim et al. 1997). Bacteria of the genera *Nitrosomonas* and *Thiobacillus* species can also dissolve phosphate compounds by producing nitric and sulphuric acids (Azam and Memon 1996).

According to the sink theory, P-solubilizing organisms **remove and assimilate P from the liquid** and hence, activate the indirect dissolution of calcium phosphate compounds by consistent removal of P from liquid culture medium. For instance, the P content in the biomass of *Pseudomonas* sp. and *P. aurantiogriseum* were similar to those observed in non-P-solubilizing microorganisms (Illmer et al. 1995) which can be explained by the fact that the P content in biomass of organisms is consistently correlated with the decomposition of P containing organic substrates (Dighton and Boddy 1989).

The other mechanism is the **production of H**_**2**_**S,** which react with ferric phosphate to yield ferrous sulphate with concomitant release of phosphate (Swaby and Sperber 1958).

(Rudolph It has been suggested that MPS activity occurs as a consequence of **microbial sulphur oxidation**^1922^), nitrate production and CO_2_ formation. These processes result in the formation of inorganic acids like sulphuric acid (Sperber 1958a). However, their effectiveness has been less accepted than the concept of involvement of organic acids in solubilization (Kim et al. 1997).

**H**^**+**^**excretion originating from NH**_**4**_^**+**^**assimilation** as proposed by Parks et al. (1990) could be the alternative mechanisms of P solubilization. An HPLC analysis of the culture solution of *Pseudomonas* sp., in contrast to the expectation, did not detect any organic acid while solubilization occurred (Illmer and Schinner 1995). They also reported that the most probable reason for solubilization without acid production is the release of protons accompanying respiration or NH_4_^+^ assimilation. Krishnaraj et al. (1998) have proposed a model highlighting the importance of protons that are pumped out of the cell to be the major factor responsible for P solubilization. Here direct role of organic or inorganic acids has been ruled out. For some microorganisms, NH_4_^+^ driven proton release seems to be the sole mechanism to promote P solubilization. Asea et al. (1988) tested two fungi, *Penicillium bilaii* and *Penicillium fuscum*, for their ability to solubilize phosphate rock in the presence of NH_4_^+^ or without N addition, and showed that only *P. bilaii* maintained the ability to decrease the pH and mobilize P when no N was supplied. In a study of *Pseudomonas fluorescens*, the form of C supply (e.g. glucose versus fructose) rather than N supply (e.g. NH_4_^+^ versus NO_3_^-^) had the greatest effect on proton release (Park et al. 2009). Further, the involvement of the H^+^ pump mechanism in the solubilization of small amounts of P in *Penicillium rugulosum* is reported (Reyes et al. 1999). Acidification of the rhizosphere of cactus seedlings (giant cardon, *Pachycereus pringlei*) after inoculation with the plant growth-promoting bacterium *Azospirillum brasilense*, in the presence or absence of ammonium and nitrate, was studied and it was assumed that the effect of inoculation with this PGPB on plant growth, combined with nitrogen nutrition, might be affecting one or more of the metabolic pathways of the plant which increases proton efflux from roots and liberation of organic acid, leading to rhizosphere acidification (Carrillo et al. 2002).This indicates that for different species, different mechanisms are responsible for proton release, only partly depending on the presence of NH_4_^+^.

Goldstein (1995) suggested that **extracellular oxidation via direct oxidation pathway** may play an essential role in soils where calcium phosphates provide a significant pool of unavailable mineral phosphorus. This has been confirmed by some researchers (Song et al. 2008) by biochemical analysis of lowering of pH in insoluble P solubilization by *Burkholderia cepacia* DA23.

**B) Organic P solubilization** is also called mineralization of organic phosphorus. Mineralization of soil organic P (Po) plays an imperative role in phosphorus cycling of a farming system. Organic P may constitute 4–90% of the total soil P (Khan et al. 2009b). Such P can be released from organic compounds in soil by enzymes:

(i) **Non-specific acid phosphatases (NSAPs**), which dephosphorylate phospho-ester or phosphoanhydride bonds of organic matter. Among the variety of phosphatase enzyme classes released by PSM, phosphomonoesterases (often just called phosphatases) are the most abundant and best studied (Nannipieri et al. 2011). Depending on their pH optima, these enzymes are divided into acid and alkaline phosphomonoesterases and both can be produced by PSM depending upon the external conditions (Kim et al. 1998; Jorquera et al. 2008). Typically, acid phosphatases predominate in acid soils, whereas alkaline phosphatases are more abundant in neutral and alkaline soils (Eivazi and Tabatabai 1977; Juma and Tabatabai 1977, 1998; Renella et al. 2006). Although plant roots can produce acid phosphatases they rarely produce large quantities of alkaline phosphatases, suggesting that this is a potential niche for PSM (Juma and Tabatabai 1998; Criquet et al. 2004). It is also difficult to differentiate between root- and PSM-produced phosphatases (Richardson et al. 2009a, b) but some evidence suggests that phosphatases of microbial origin possess a greater affinity for Po compounds than those derived from plant roots (Tarafdar et al. 2001). The relationship between PSM introduced into soil, phosphatase activity and the subsequent mineralization of Po still remains poorly understood (Chen et al. 2003)
(ii) **phytases**, which specifically cause release of P from phytate degradation. In its basic form, phytate is the primary source of inositol and the major stored form of P in plant seeds and pollen, and is a major component of organic P in soil (Richardson, 1994). Although the ability of plants to obtain P directly from phytate is very limited, yet the growth and P-nutrition of Arabidopsis plants supplied with phytate was significantly improved when they were genetically transformed with the phytase gene (phyA) derived from *Aspergillus niger* (Richardson et al. 2001). This led to an increase in P-nutrition to such an extent that the growth and P-content of the plant was equivalent to control plants supplied with inorganic P. Hence microorganisms are in fact a key driver in regulating the mineralization of phytate in soil and their presence within the rhizosphere may compensate for a plants inability to otherwise acquire P directly from phytate (Richardson and Simpson 2011).
(iii) **phosphonatases and C–P lyases,** that cleave the C–P bond of organophosphonates (Rodriguez et al. 2006).

**Figure 2:**
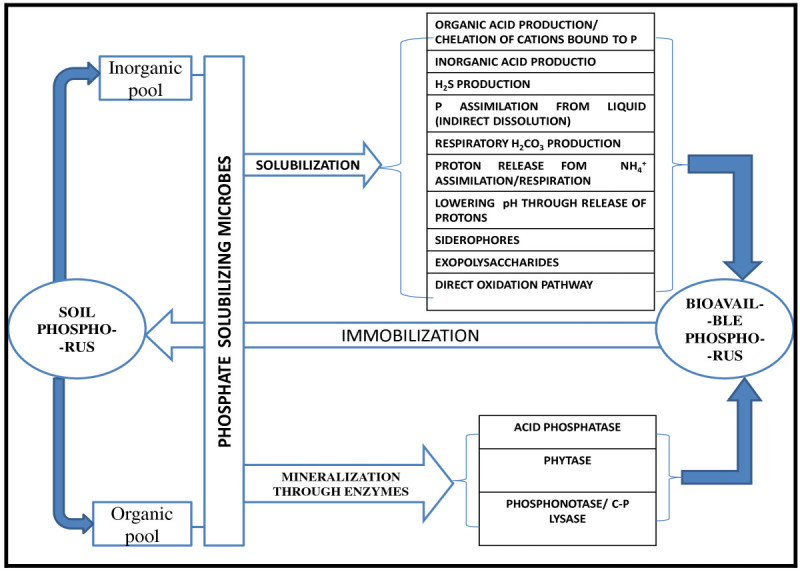
**Schematic representation of mechanism of soil P solubilization/mineralization and immobilization by PSM.**

It is therefore clear that P solubilization by PSMs has been a subject of analysis and research for a long time and still the research seems to be in its infancy. It occurs through different mechanisms and there is considerable variation amongst the organisms in this respect. Each organism can act in one or more than one way to bring about the solubilization of insoluble P. Though it is difficult to pin point a single mechanism, production of organic acids and consequent pH reduction appears to be of great importance. Different mechanisms involved in the solubilization and mineralization of insoluble P by naturally-occurring microbial communities of soils are briefly illustrated in Figure 2.

### Role of siderophores in P solubilization

Siderophores are complexing agents that have a high affinity for iron and are produced by almost all microorganisms in response to iron deficiency. Thus siderophores act as solubilizing agents for iron from minerals or organic compounds under conditions of iron limitation. There are approximately 500 known siderophores, with the majority of them being used by a wide range of microorganisms and plants and some of them being exclusively used by the microbial species and strains that produce them (Crowley 2007). Studies have reported the release of siderophores from PSM (Vassilev et al. 2006; Caballero-Mellado et al. 2007; Hamdali et al. 2008a); however, siderophore production has not been widely implicated as a P-solubilization mechanism. Considering the dominance of mineral dissolution over ligand exchange by organic acid anions as a P-solubilizing mechanism (Parker et al. 2005), the potential role of siderophores in enhancing P availability should be obvious.

### Role of EPS in P solubilization

Recently the role of **polysaccharides** in the microbial mediated solubilization of P was assessed by Yi et al. (2008). Microbial exopolysaccharides (EPSs) are polymers consisting mainly of carbohydrates excreted by some bacteria and fungi onto the outside of their cell walls. Their composition and structures are very varied; they may be homo- or heteropolysaccharides and may also contain a number of different organic and inorganic substituents (Sutherland 2001). Four bacterial strains of *Enterobacter sp. (EnHy-401), Arthrobacter sp. (ArHy-505), Azotobacter sp. (AzHy-510) and Enterobacter sp. (EnHy-402),* possessing the ability to solubilize TCP (tri calcium phosphate), were used to assess the role of exopolysaccharide (EPS) in the solubilization of P by Yi et al. 2008. These Phosphate Solubilizing bacteria produced a significant amount of EPS and demonstrated a strong ability for P-solubilization. However further studies are necessary to understand the relationship between EPS production and phosphate solubilization.

### Plant growth promoting attributes of PSM

**Figure 3:**
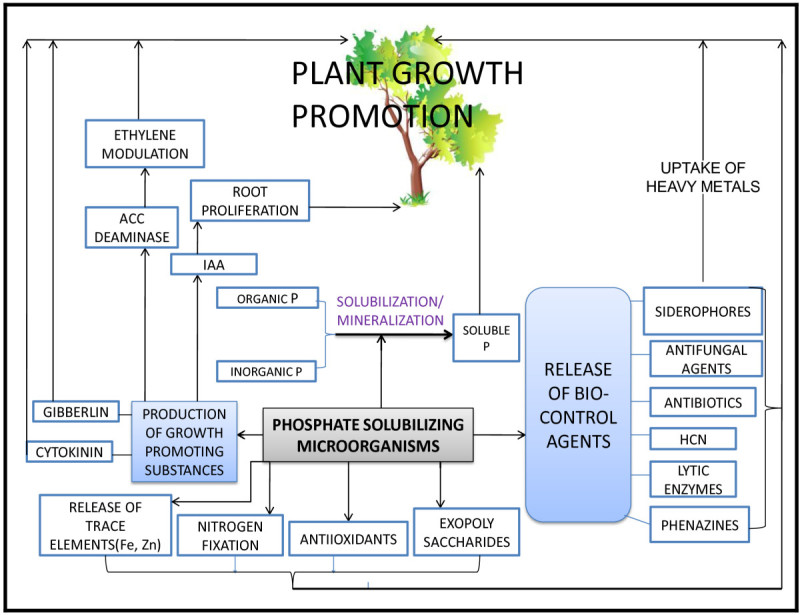
**Possible Mechanisms involved in plant growth promotion by PSM.**

Besides making soluble P accessible for uptake by plants, there have been a number of reports on plant growth promotion by these microorganisms (Gaur and Ostwal 1972). This is achieved by production of plant beneficial metabolites, such as phytohormones, antibiotics, or siderophores. Various PSM preparations have been shown to promote the growth of many crops (Table 3). Endophytic Bacteria isolated from rhizoplane of cacti growing in bare lava rocks, not only significantly mobilized Phosphate and other minerals (Puente et al. 2004a, 2009a) but also promoted growth of wild cactus species (Puente et al. 2004b, b). The mechanisms involved in plant growth promotion by PSM are outlined in Figure 3.

**Table 3:** **Plant growth promotion by PSM (Patil et al.** 2002 **)**

PSM Bioinoculant	Crop benefited
*B. firlmus* NCIM 2636	Paddy in acid soils
*G. faciculatum*	Banana
*B. megaterium*+*G. faciculatum*	Banana
Phosphobacterium	Sword bean variety SBS 1
*P. Striata*	Soybean in sandy alluvial soil
*P. Striata*	Chick pea

### Genetic engineering of PSM

Although knowledge of the genetics of phosphate solubilization is still scanty, and the studies at the molecular level in order to understand how precisely the PSM brings out the solubilization of insoluble P are inconclusive (Rodriguez et al. 2006). However, some genes involved in mineral and organic phosphate solubilization have been isolated and characterized. Initial achievements in the manipulation of these genes through genetic engineering and molecular biotechnology followed by their expression in selected rhizobacterial strains open a promising perspective for obtaining PSM strains with enhanced phosphate solubilizing capacity, and thus, a more effective use of these microbes as agricultural inoculants. The initial achievement in cloning of gene involved in P solubilization from the Gram negative bacteria *Erwinia herbicola* was achieved by Goldstein and Liu (1987). Similarly the napA phosphatase gene from the soil bacterium *Morganella morganii* was transferred to *Burkholderia cepacia IS-16*, a strain used as a biofertilizer, using the broad-host range vector pRK293 (Fraga et al. 2001). An increase in extracellular phosphatase activity of the recombinant strain was achieved.

Introduction or over-expression of genes involved in soil phosphate solubilization (both organic and inorganic) in natural rhizosphere bacteria is a very attractive approach for improving the capacity of microorganisms to work as inoculants. Insertion of phosphate-solubilizing genes into microorganisms that do not have this capability may avoid the current need of mixing two populations of bacteria, when used as inoculants (nitrogen fixers and phosphate-solubilizers (Bashan et al. 2000). There are several advantages of developing genetically-modified PSM over transgenic plants for improving plant performance: (1) With current technologies, it is far easier to modify a bacterium than complex higher organisms, (2) Several plant growth-promoting traits can be combined in a single organism, and (3) Instead of engineering crop by crop, a single, engineered inoculant can be used for several crops, especially when using a non-specifc genus like Azospirillum (Rodriguez et al. 2006). Some barriers should be overcome first to achieve successful gene insertions using this approach, such as the dissimilarity of metabolic machinery and different regulating mechanism between the donor and recipient strains. Despite the diffculties, significant progress has been made in obtaining genetically engineered microorganisms for agricultural use (Armarger 2002). Overall, further studies on this aspect of PSM will provide crucial information in future for better use of these PSM in varied environmental conditions.

### Current trends

Phosphorus is an important limiting factor in agriculture production, and considering the negative effects of chemical P fertilizers, microbial intervention of PSM seems to be an effective way to solve the phosphorus availability in soil. However P-solubilization in soil is much more difficult to study than solubilization of P in broth culture. The crops respond differently to the inoculation of PSMs and are dependent on several factors such as the soil temperature, moisture, pH, salinity, and source of insoluble P, method of inoculation, the energy sources and the strain of microorganism used. Hence study of PSM activity in correlation with these factors has to be done extensively before PSM can be used as a biofertiliser with promising results. The successful implementation of this approach has already been demonstrated in the fields by various workers, to a limited extent. However the large scale use of this technology would benefit from additional studies, particularly those directed towards understanding how the interaction between soil and microbial system might be facilitated.

The organisms involved in phosphorus (P) cycling in soils are highly varied, and microorganisms probably play the most important role. However, more than 99% of soil microorganisms have not been cultured successfully (Torsvik and Ovreas 2002). Therefore, culture-independent methods are required to study the function and ecology of microbes involved in P cycling in soils. Molecular approaches for such culture-independent methods have been developed in the recent past. The molecular techniques based on nucleic acid composition like LMW RNA profiling and PCR based techniques, are excellent tools for this purpose, as they are precise, reproducible and not dependent on culture media composition or growth phase of microorganisms (Peix et al. 2007).

Molecular-based techniques also provide new opportunity to detect the presence and abundance of specific microorganisms or to quantify the expression of target genes directly in soil or in the rhizosphere with high levels of sensitivity. For example, specific primers based on conserved regions have been described for various microorganisms associated with P mobilization, including mycorrhizal fungi, Penicillium sp., and Pseudomonas sp. (Oliveira et al. 2009), as have primers that are directed at traits such as bacterial phytases (Jorquera et al. 2011). Microarrays composed of suites of functional bacterial genes and arrays for phylogenetic analysis of bacterial diversity based on 16S-RNAgene sequences along with next-generation sequencing and soil microbiome analyses; provide further application for assessment of diversity surrounding particular traits or functional groups of microorganisms (Richardson and Simpson 2011). Collectively, these tools provide new opportunities to address key questions in microbial community ecology and to assess the survival and persistence of specific inoculants under different environmental conditions.

Looking at the possible avenues which can open up with exploring these environmental friendly microorganisms, it is necessary to study the composition and dynamics of these microbial populations to reach a better understanding of soil PSM diversity and P uptake by plants.

### Future prospects

Despite their different ecological niches and multiple functional properties, P-solubilizing microorganisms have yet to fulfill their promise as commercial bio-inoculants. Current developments in our understanding of the functional diversity, rhizosphere colonizing ability, mode of actions and judicious application are likely to facilitate their use as reliable components in the management of sustainable agricultural systems. Although significant studies related to PSM and their role in sustainable agriculture have been done over the last few decades, the required technique remains in its infancy. Nevertheless with an awareness of the limitations of existing methods, a reassessment can be expected, so that the use of PSM as potential biofertilisers in different soil conditions becomes a reality.

Enhancement in the use of PSM is one of the newly emerging options for meeting agricultural challenges imposed by the still-growing demand for food. Thus, more than ever, obtaining high yields is the main challenge for agriculture. In addition, in recent years both producers and consumers have increasingly focused on the health and quality of foods, as well as on their organoleptic and nutritional properties. Hence, this biotechnology is also likely to ensure conservation of our environments. However, before PSM can contribute to such benefits, scientists must learn more about them and explore ways and means for their better utilization in the farmers’ fields. Future research should focus on managing plant–microbe interactions, particularly with respect to their mode of actions and adaptability to conditions under extreme environments for the benefit of plants. Furthermore, scientists need to address certain issues, like how to improve the efficacy of biofertilizers, what should be an ideal and universal delivery system, how to stabilize these microbes in soil systems, and how nutritional and root exudation aspects could be controlled in order to get maximum benefits from PSM application. Biotechnological and molecular approaches could possibly develop more understanding about PSM mode of actions that could lead to more successful plant–microbe interaction. Efforts should also be directed towards the use of PSM to reduce pesticide applications. In brief, PSM biotechnology provides an excellent opportunity to develop environment-friendly phosphorus biofertilizer to be used as supplements and/or alternatives to chemical fertilizers.

## Conclusions

Phosphorus is a vital element in crop nutrition. Adverse environmental effects of chemical based P fertilsers, depleting resources of high grade Phosphatic rocks and their skyrocketing prices have compelled us to find a sustainable approach for efficient P availability in agriculture to meet the ever increasing global demand of food. Soil microorganisms are involved in a range of processes that affect P transformation and thus influence the subsequent availability of P (as phosphate) to plant roots. In particular, microorganisms can solubilize and mineralize P from inorganic and organic pools of total soil P.

The use of efficient PSM (phosphate-solubilizing microorganisms), opens up a new horizon for better crop productivity besides sustaining soil health. However, the viability and sustainability of PSM technology largely depends on the development and distribution of good quality inoculants to farming communities. Therefore, there is a need for extensive and consistent research efforts to identify and characterize more PSM with greater efficiency for their ultimate application under field conditions. Soil Scientists and Microbiologists have a great responsibility to the society to find ways and means as to how soil P could be improved without applying the chemical P fertilizers under different agro-climatic regions of the world.

The promise of exploiting soil microorganisms to increase mobilization of soil P remains. Whether or not this will be achieved through better management of soil microbial communities, by development of more effective microbial inoculants, through the genetic manipulation of specific organisms, or with a combination of these approaches is not known. What is clear though is that soil microorganisms play an important role in the mobilization of soil P and that detailed understanding of their contribution to the cycling of P in soil-plant systems is required for the development of sustainable agriculture and our movement from a green revolution to an evergreen revolution can be accompolished.

## Authors’ information

Author SBS is the Principal Investigator of a project entitled “Study of the microbiological diversity in different agricultural soils of kachchh with special reference to Phosphate Solubilising Microbes”. The project is funded by the Women Scientist Scheme (WOS-A) of Department Of Science And Technology, Government of India, New Delhi. Under this project agricultural fields applying different amendments are studied for their microbial diversity as well as physico chemical properties in various seasons and any correlation which, if exists is being studied. The insights gained through this study will help to understand the microbial diversity in this very unique ecological zone of Kachchh, Gujarat, Western India.

## Authors’ original submitted files for images

Below are the links to the authors’ original submitted files for images. Authors’ original file for figure 1 Authors’ original file for figure 2 Authors’ original file for figure 3
