# Supplementary material for: Phosphate solubilizing microbes: sustainable approach for managing phosphorus deficiency in agricultural soils
Source: Springerplus. 2013 Oct 31;2:587. doi: 10.1186/2193-1801-2-587 (PMC4320215; doi:10.1186/2193-1801-2-587)
Supplement: Supplementary file 1 — Authors’ original file for figure 1 [file 40064_2013_1439_MOESM1_ESM.pdf]

Soil sample collection

↓

Serial dilution

↓

Inoculation on media (pour plate/streaking) having different sources of insoluble P, depending on soil type

↓

Clear zone around colony growth indicates PSM activity

↓

Additional test in liquid media to assay P dissolution

↓

Test isolates for abundant production of organic acids

↓

Pure cultures by reinoculation

↓

Study of the morphology and colony characteristics and biochemical tests

↓

Screening of best inoculants in terms of P solubilizing activity

↓

Identification at genetic level (molecular characterization)

↓

Test on a model plant

↓

Development of microphos

↓

Green house trials

↓

Field trials

↓

Standardization (quality control)

↓

Commercial biofertiliser
