# Supplementary figures and images for: Phosphate solubilizing microbes: sustainable approach for managing phosphorus deficiency in agricultural soils
Source: Springerplus. 2013 Oct 31;2:587. doi: 10.1186/2193-1801-2-587 (PMC4320215; doi:10.1186/2193-1801-2-587)

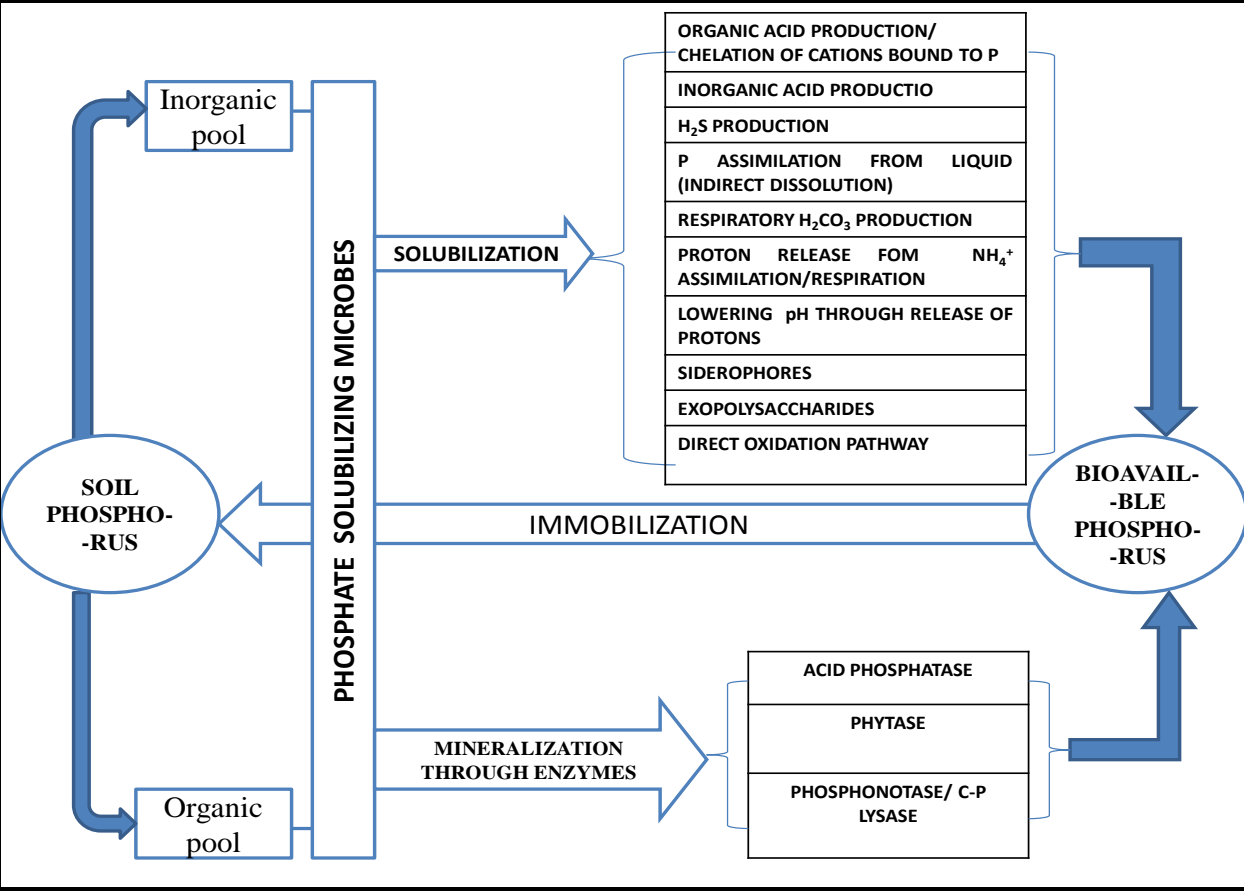

Supplement: Supplementary file 2 — Authors’ original file for figure 2 [file 40064_2013_1439_MOESM2_ESM.pdf]

# PLANT GROWTH PROMOTION

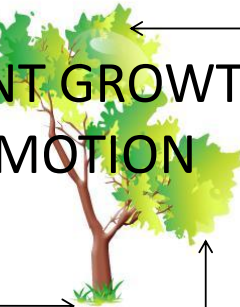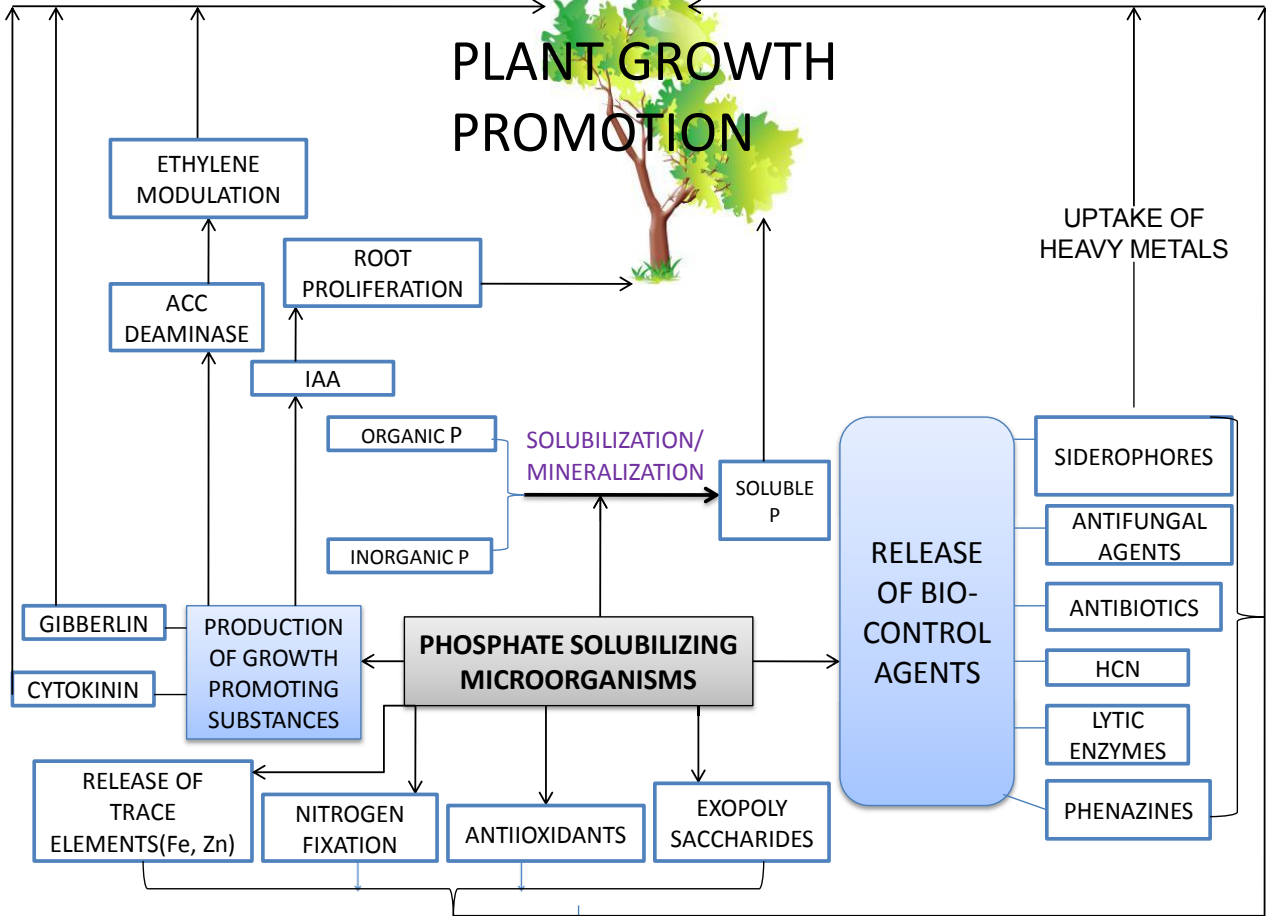

Supplement: Supplementary file 3 — Authors’ original file for figure 3 [file 40064_2013_1439_MOESM3_ESM.pdf]
